# The U-Shaped Association between Bilirubin and Diabetic Retinopathy Risk: A Five-Year Cohort Based on 5323 Male Diabetic Patients

**DOI:** 10.1155/2018/4603087

**Published:** 2018-10-30

**Authors:** Miao Liu, Jianhua Wang, Yao He

**Affiliations:** Institute of Geriatrics, Beijing Key Laboratory of Aging and Geriatrics, National Clinical Research Center for Geriatrics Diseases, State Key Laboratory of Kidney Diseases, Chinese PLA General Hospital, 28 Fuxing Road, Beijing 100853, China

## Abstract

**Aims:**

This study aimed at assessing the impact of baseline bilirubin (TBiL) on the incidence of diabetic retinopathy (DR) based on a five-year cohort study which consisted of 5323 Chinese male diabetic patients.

**Methods:**

A cohort study based on 5323 male diabetic patients was conducted in Beijing, from 2009 to 2013. Both baseline TBiL and follow-up changes were measured. Cox proportional risk model was used to calculate the hazard ratio (HR) of TBiL for DR risk.

**Results:**

During the follow-up period, there were 269 new DR cases. The incidence of five-year follow-up was 5.1% (95% CI: 4.5%~5.6%). The TBiL level of those who had diabetic retinopathy was lower than that of those without (12.51+ 1.20 mol/L and 13.11+ 1.32 *μ*mol/L, *P* = 0.033). And more interestingly, along with the quintiles of baseline TBiL, there showed a U-shaped curve with DR incidence. And the RRs were 0.928 (95% CI: 0.646–1.331), 0.544 (95% CI: 0.365–0.811), 0.913 (95% CI: 0.629–1.324), and 1.035 (95% CI: 0.725–1.479) for the second, third, fourth, and fifth quintiles of baseline TBiL levels, respectively, compared with the first quintile. For follow-up TBiL changes, after being adjusted for related covariables and baseline TBiL levels (as continuous variable) in the model, the RRs for DR were 1.411 (95% CI: 1.081–1.842) for those who had decreased TBiL level and 0.858 (95% CI: 0.770–0.947) for those who had increased TBiL level during follow-up. And this association was more prominent among those with lower baseline TBiL level.

**Conclusions:**

Serum TBiL had a U-shaped relationship with DR incidence, which was independent of control status of diabetes and other related covariates.

## 1. Introduction

Diabetic retinopathy (DR) is one of the important vascular complications of diabetes. Data show that this is the main cause of blindness among working age population in developing countries [1]. Therefore, exploring the pathogenesis of DR is of most importance [2]. Previous basic and clinical research data indicated that oxidative stress played an important role in the development of DR.

Total bilirubin (TBIL) has been considered as a powerful endogenous antioxidant in recent years. A number of studies have shown that elevated TBiL levels were negatively correlated with cardiovascular disease and diabetes mellitus [3–5]. In addition, a number of studies have reported a protective relationship between TBiL levels and diabetic vascular complications. There was also a meta-analysis of the association between TBiL and DR published in 2016 [6]. However, these studies were mostly cross-sectional or case-control studies with small sample sizes and inconsistent findings. Some studies show that there was no association between TBiL and DR risk [7, 8]. In addition, there was little evidence of dose-response effects, which was of great value in determining appropriate clinical thresholds of TBiL levels among diabetic patients. Therefore, based on this five-year cohort study with a large sample (more than 5000 elderly diabetic patients), our study assessed the relationship between TBIL and its changes and the incidence of DR.

## 2. Methods

### 2.1. Subjects

**Figure 1:**
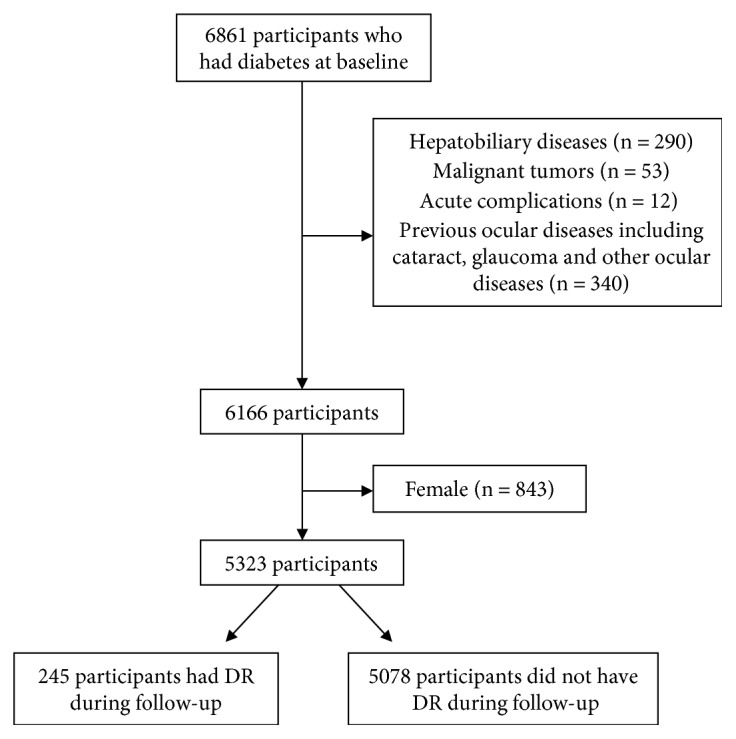
Flow chart of inclusion of participants.

This cohort study consisted of elderly diabetic patients. We conducted the baseline survey in 2009, and a total of 6861 elderly were recruited, and the follow-up survey was done in 2013. The details were described in a previous article [9]. Considering the possible effects of related diseases on TBiL or diabetes, we excluded those patients who had hepatobiliary diseases (*n* = 290), malignant tumors (*n* = 53), acute diabetic complications such as ketoacidosis (*n* = 12), previous ocular diseases including cataract, glaucoma, and other ocular diseases (*n* = 340). Considering female just accounted for 13.7% (843) of the total participants, only 5323 male participants were left for further analysis.  Figure 1 shows the flow chart of participants' inclusion.

### 2.2. Data Collection

Baseline anthropometric and physical examination information was collected according to the standard process. Fasting blood was collected, and the related biochemical indexes were detected. The details were described in a previous article [9].

### 2.3. Definitions

The duration of diabetes was calculated by age minus the age of first diagnosis on diabetes. Quintiles of baseline TBiL level were defined as follows: Q1: ≤9.20 *μ*mol/L, Q2: 9.20–12.60 *μ*mol/L, Q3: 12.60–13.80 *μ*mol/L, Q4: 13.80–16.50 *μ*mol/L, and Q5: ≥16.50 *μ*mol/L. Follow-up changes of TBiL were defined as follow-up TBiL levels minus baseline TBiL levels and were divided into three categories: ≤−2 *μ*mol/L; −2 to 2 *μ*mol/L; and ≥2 *μ*mol/L. Diabetes, hypertension, and dyslipidemia were defined according to the corresponding guidelines. DR was defined according to the Chinese version of guidelines for the prevention and treatment of type 2 diabetes: the presence of mild or moderate or proliferative retinopathy in either eye [10], and was independently diagnosed by two senior ophthalmologists.

### 2.4. Statistical Analysis

For continuous data, mean *±* SD was used for description, and analysis of variance was used for comparisons. For categorical variables, chi-square test is used for comparison. Multivariate Cox proportional hazard model was used to estimate the hazard ratio (HR) and 95% confidence intervals (CIs) of DR based on baseline TBiL levels and follow-up TBiL changes. Restricted cubic spline functions with 5 knots were used to test the potential nonlinear association and display the data graphically. SPSS software was used for data analysis. *P* < 0.05 was statistically significant.

### 2.5. Ethical Consideration

The ethics committee of the General Hospital of PLA approved the study (EC0411-2001). Each participant signed a written informed consent.

## 3. Results

### 3.1. Baseline Characteristics according to DR Incidence

Table 1 shows the baseline characteristics of the subjects. The mean age of the 5323 diabetic patients was 78.68 ± 8.39 (65~102 yrs). Mean diabetes duration and TBiL level were 17.25 ± 7.49 yrs and 13.08 ± 1.32 *μ*mol/L. The percentage of overweight/obesity, hypertension, and dyslipidemia was 68.4%, 62.4%, and 35.8%, respectively. Compared with those who did not have DR, those with DR had relatively longer duration, higher FPG and 2hPG levels, and lower baseline TBiL level (*p* < 0.05). Baseline characteristics according to quintiles of baseline TBiL levels were presented in Appendix Table 1. Along with the increase of baseline TBiL levels, it showed shorter duration; higher Hb, HDL-C, FPG, and 2hPG levels; and lower percentage of diabetes control status.

### 3.2. Incidence of DR according to Baseline TBiL Quintiles and Follow-Up TBiL Changes

There were a total of 269 DR cases during the 21,586 person-years. The total five years' incidence was 5.1% (95% CI: 4.5%–5.6%). As we can see from Table 2, the incidence of the quintiles of baseline TBiL for DR had fluctuations; the third quintile had the lowest incidence while the first and the fifth had the highest incidence. The incidence density showed a similar trend. The incidence of follow-up TBiL changes for DR was lowest among those who had an increase of ≥2 *μ*mol/L while highest among those who had a decrease of ≤−2 *μ*mol/L (Table 2).

### 3.3. HRs and 95% CI of DR Incidence according to Baseline TBiL Levels

**Figure 2:**
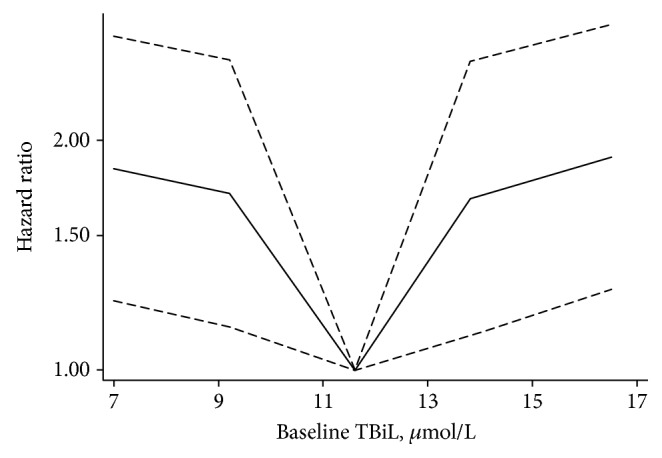
HRs of follow-up TBiL changes for DR incidence according to baseline TBiL level.

Table 3 showed the HRs of baseline TBiL levels for DR incidence. We adjusted related covariables according to results of univariate analysis (Appendix Table 1); the HRs of baseline TBiL levels for DR were 0.9656 (95% CI: 0.934–0.983). When quintiles were used as categorical variables, the HRs were 1.838 (95% CI: 1.233–2.740), 1.705 (95% CI: 1.138–2.555), 1.678 (95% CI: 1.109–2.540), and 1.903 (95% CI: 1.275–2.841) for the first, second, fourth, and fifth quintiles of baseline TBiL levels, respectively, compared with the third quintile. And restricted cubic spline functions depicted a sort of U-shaped curve (Figure 2).

### 3.4. HRs for DR Incidence according to Follow-Up TBiL Changes

The follow-up TBiL level was lower than the baseline TBiL level. The mean level of the baseline TBiL was 13.05 ± 1.65 (median: 12.30, IQR: 9.20–15.30) *μ*mol/L, and the mean follow-up TBiL level was 12.54 ± 1.64 (median: 12.50, IQR: 10.00–15.70) *μ*mol/L; thereby, the follow-up TBiL changes were −0.66 ± 1.93 (median: −0.73, IQR: −3.30 to 2.10) *μ*mol/L (Appendix Table 2). Those who had DR in the follow-up years had a relatively bigger TBiL change; the mean follow-up TBiL changes were − 1.60 ± 1.48 (median: −1.11, IQR: −3.86 to 1.20) *μ*mol/L.

For follow-up TBiL changes, after being adjusted for related covariables and baseline TBiL levels in the model, the HRs for DR were 0.967 (95% CI: 0.944–0.991). When used as categorical variables, as we can see from Table 4, compared with those who had relatively stable TBiL levels (−2 *μ*mol/L < follow-up TBiL changes < 2 *μ*mol/L), the HR for DR incidence was higher (HR = 1.411, 95% CI: 1.081–1.842) among those who had follow-up TBiL changes ≥2 *μ*mol/L, and the HR was lower (HR = 0.858, 95% CI: 0.770–0.947) among those with follow-up TBiL changes ≤−2 *μ*mol/L. Besides, the decreasing trend of follow-up TBiL changes for DR incidence was more obvious among those with lower baseline TBiL level (≤12.5 *μ*mol/L, *n* = 2669). However, among those with relatively higher baseline TBiL level (>12.5 *μ*mol/L, *n* = 2654), the HRs of follow-up TBiL changes showed no significant results.

In the sensitivity analysis, when participants who had DR that happened within less than 2 years were excluded (*n* = 37) or divided by age groups (≤80 yrs and >80 yrs), the trend of adjusted HRs was similar with the results from Tables 3 and 4 (Appendix Tables 3, 4, 5, and 6).

## 4. Discussion

In this study, we did a deep study and evaluated the relationship between baseline TBiL and DR incidence using a large cohort of more than 5000 male elderly. The results showed that baseline TBiL had a U-shaped relationship with DR incidence, rather than a simple linear relationship. And this association was independent of control status of diabetes and other related covariates. And more interestingly, increased follow-up TBiL changes had a higher DR risk, and this association was more prominent among those with lower baseline TBiL level.

There were several researches focused on relationships between TBiL level and DR risk [11, 12], even one meta-analysis published in 2016 [6]. Most of the previous studies showed that there was a negative relationship between TBiL and DR. However, the result of our deep study showed that the association between TBiL and DR risk was not a simple linear association but a U-shaped curve. From the meta-analysis of TBiL and DR, we could see that most studies were cross-sectional ones or case-control studies with a small sample. Our study was based on a five-year cohort which consisted of more than 5000 elderly diabetic patients. And this U-shaped curve was consistent with previous ones about relationships about TBiL with cardiovascular diseases. A cohort study based on 7685 middle-aged British men firstly revealed that there was a U-shaped relationship between TBiL and risk of ischemic heart disease [13]. And in 2012, results from one of the biggest cohorts based on more than 130,000 patients who received statin treatment showed that a U-shaped association appeared between TBiL before statin prescription and coronary heart diseases [14]. Our result was consistent with these prospective studies [15]. And this implies that the DR risk would no longer decrease when baseline TBiL increased to a relatively higher level. Meanwhile, there were a series of evidences showing that a higher TBiL level (beyond normal range) indicated hepatocellular injuries, and the latter one was proved to be associated with increased risk of diabetes and cardiovascular disease [7]. Besides, the negative relationship might be part of the U-shaped curve, since most of the cross-sectional studies were of small sample size. And the actual U-shaped association might be the combination of antioxidation and liver toxicity effects.

The pathogenesis of DR has not been fully studied. Oxidative stress caused by high glucose is one of the hotspots of the current research [16]. TBiL, not only a metabolite of hemoglobin, is also considered to be an important endogenous antioxidant. Past studies have shown that TBiL has important protective effects on cardiovascular disease, diabetes, and diabetic macrovascular complications, mainly through anti-inflammatory and antioxidant effects [17, 18]. For DR, studies have revealed that TBiL has significant protective effects [6]. And results from the first known human case of heme oxygenase-1 deficiency showed that TBiL at physiological concentration has strong antioxidant activity, which can prevent low-density lipoprotein lipid peroxidation, and results based on mice showed that 1 mol/L direct bilirubin can remove about 2 mol/L free radicals [19]. All these evidences support the protective effect of TBiL on DR. However, the premise is in the normal range. If the TBiL was beyond the normal range, it is most probably an indicator of liver damage, which was an important risk factor for diabetes and its complications. There were several prospective studies with large sample size including our study which have showed the protective effect of TBiL in the normal range and the harmful effect in the relative higher range for cardiovascular disease and diabetes (including its complications) [14, 15, 20], which confirmed the U-shaped curve of TBiL for DR in our study. This implies that in clinical practice, the previous “higher TBiL indicates lower risk of complications” was not always right. Doctors should also pay enough attention to the abnormal range and whether there were liver toxicity effects.

Follow-up changes of TBiL also had protective effects on DR incidence, which indicated that medical workers should also pay attention to both the baseline and the fluctuation of TBiL levels among diabetic patients, and it will play an important role in predicting DR incidence. Most of the previous studies only assessed the relationship between baseline TBiL level and DR; however, only a few studies focused on the follow-up volatility. In our study, for those who had increased TBiL changes (≥2 *μ*mol/L), the risk of DR incidence had increased about 40%. And the more obvious decreasing trend among those with relatively lower baseline TBiL levels verified previous results about the U-shaped curve of the association between TBiL and DR risk.

As far as we know, this was the first study to analyze the relationship between TBiL and DR based on a large sample cohort in fully adjusted models. In addition, besides baseline TBiL levels, TBiL changes also had an independent and inverse impact on DR incidence. The cohort had rigorous investigation process, strict training for all the staff in the field survey, and high response rate because of the well-controlled follow-up system.

However, this study had the following limitations. First, TBiL was measured only once at either baseline or follow-up, so it could not reflect the actual fluctuations. Second, only TBiL was collected; there was no specific value for direct and indirect bilirubin levels, so it was impossible to distinguish the exact role of these two types. Third, all participants were retired male, whose economic and medical security was relatively good, and the representation was limited for the general population.

In summary, this large-sample cohort study had shown that TBiL and its changes in diabetic patients were independently associated with DR incidence, and this association was independent of both classical risk factors and diabetes control status. Clinical medical staff should pay attention to the monitoring of TBiL levels in order to early detect and better control DR.

## Figures and Tables

**Table 1:** General characteristics of participants according to DR incidence.

Characteristics	NDR (*n* = 5078)	DR (*n* = 245)	*P*	Total (*n* = 5323)
Mean ± SD				
Age (yrs)	78.68 ± 8.41	78.95 ± 7.80	0.623	78.68 ± 8.39
Duration (yrs)	17.14 ± 7.51	19.81 ± 7.40	0.042	17.25 ± 7.49
Height (cm)	169.64 ± 5.41	169.22 ± 5.18	0.235	169.62 ± 5.40
Weight (kg)	72.61 ± 8.53	72.66 ± 8.51	0.927	72.61 ± 8.53
BMI (kg/m^2^)	25.24 ± 2.79	25.38 ± 2.80	0.446	25.24 ± 2.79
SBP (mmHg)	133.28 ± 14.04	133.77 ± 13.64	0.593	133.30 ± 14.02
DBP (mmHg)	73.88 ± 9.46	72.68 ± 8.97	0.052	73.82 ± 9.44
Hb (g/L)	132.11 ± 15.44	131.16 ± 20.37	0.848	132.07 ± 17.83
TC (mmol/l)	4.75 ± 1.05	4.68 ± 1.00	0.307	4.75 ± 1.05
TG (mmol/l)	1.68 ± 1.08	1.59 ± 1.00	0.196	1.67 ± 1.07
HDL-C (mmol/l)	1.33 ± 0.44	1.23 ± 0.32	<0.001	1.33 ± 0.44
LDL-C (mmol/l)	2.67 ± 0.83	2.74 ± 0.83	0.186	2.67 ± 0.83
FPG (mmol/l)	6.93 ± 1.89	6.95 ± 1.92	0.046	6.94 ± 1.90
2hPG (mmol/l)	9.24 ± 2.83	9.96 ± 2.35	<0.001	9.27 ± 2.86
ALT (U/L)	20.81 ± 4.59	19.91 ± 4.99	0.041	20.76 ± 4.68
Baseline TBiL (*μ*mol/L)	13.11 ± 1.32	12.51 ± 1.20	0.033	13.08 ± 1.32
%				
Education			0.105	
≤6 yrs	65.1	65.3		65.4
≥7 yrs	34.9	34.7		34.6
Marriage status			0.077	
Divorced/widowed	16.5	12.3		12.7
Married	87.5	83.7		87.3
Current smoking			0.186	
Yes	21.7	18.6		21.5
No	78.3	81.4		78.5
Current alcohol drinking			0.854	
Yes	19.7	19.5		18.7
No	80.3	80.5		80.3
Overweight/obesity			0.640	
Yes	68.4	69.8		68.4
No	31.6	30.2		31.6
Hypertension			0.171	
Yes	62.2	66.5		62.4
No	37.8	33.5		37.6
Dyslipidemia			0.101	
Yes	34.8	35.3		35.8
No	65.2	64.7		64.2
Control of diabetes			0.907	
Yes	50.7	50.5		50.8
No	49.3	49.5		49.2

Data are mean ± SD for continuous values or % for category values.

**Table 2:** Incidence of DR according to baseline TBiL quintiles and follow-up TBiL changes.

DR	Quintiles of baseline TBiL(*μ*mol/L)					Follow-up TBiL changes (*μ*mol/L)			Total
	Q1 (≤9.20)	Q2 (9.20–12.60)	Q3 (12.60–13.80)	Q4 (13.80–16.50)	Q5 (≥16.50)	≤−2	−2 to 2	≥2	
Number of incident cases	61	57	40	51	60	115	105	49	269
Incidence (%)	5.6 (4.3–7.0)	5.4 (4.0–6.8)	3.4 (2.4–4.5)	5.2 (3.8–6.2)	5.7 (4.3–7.1)	6.3 (5.2–7.5)	4.9 (4.0–5.9)	3.7 (2.7–4.8)	5.1 (4.5–5.6)
Total person-years	4399	4302	4638	3981	4267	7443	8692	5451	21,586
Incidence density (per 100 person-years)	1.4 (1.1–1.8)	1.3 (1.0-1.7)	0.9 (0.6–1.2)	1.3 (1.0-1.7)	1.4 (1.1–1.8)	1.5 (1.3–1.9)	1.2 (1.0-1.5)	0.9 (0.7–1.2)	1.2 (1.1–1.4)

**Table 3:** HRs and 95% CI of DR incidence according to baseline TBiL levels (*μ*mol/L).

Variable type	HR^∗^ (95% CI)	*P*
Continuous variable	0.956 (0.934–0.983)	0.001
Quintiles		0.017
Q1 (≤9.20)	1.838 (1.233–2.740)	
Q2 (9.20–12.60)	1.705 (1.138–2.555)	
Q3 (12.60–13.80)	1.00 (Ref)	
Q4 (13.80–16.50)	1.678 (1.109–2.540)	
Q5 (≥16.50)	1.903 (1.275–2.841)	

^∗^Adjusted for age, marital status, current smoking, current alcohol drinking, BMI, baseline Hb, ALT, baseline prevalence of hypertension and dyslipidemia, control of diabetes, duration of diabetes, and follow-up TBiL changes (as continuous variable) in the model.

**Table 4:** HRs and 95% CI of DR incidence according to follow-up TBiL changes (*μ*mol/L).

	Variable type	HR^∗^ (95% CI)	*P*
Total population (*n* = 5323)	Continuous variable	0.967 (0.944–0.991)	0.006
	Categorical variable		0.011
	≤−2	1.411 (1.081–1.842)	
	−2 to 2	1.00 (Ref)	
	≥2	0.858 (0.770–0.947)	

Among those with baseline TBiL level ≤ 12.5 *μ*mol/L (*n* = 2669)	Continuous variable	0.964 (0.931–0.986)	0.037
	Categorical variable		0.003
	≤−2	1.756 (1.217–2.534)	
	−2 to 2	1.00 (Ref)	
	≥2	0.853 (0.761–0.934)	

Among those with baseline TBiL level > 12.5 *μ*mol/L (*n* = 2654)	Continuous variable	0.969 (0.936–1.003)	0.072
	Categorical variable		0.794
	≤−2	1.059 (0.689–1.626)	
	−2 to 2	1.00 (Ref)	
	≥2	0.864 (0.451–1.654)	

^∗^Adjusted for age, marital status, current smoking, current alcohol drinking, BMI, baseline Hb, ALT, baseline prevalence of hypertension and dyslipidemia, control of diabetes, duration of diabetes, and baseline TBiL levels (as continuous variable) in the model.

## Data Availability

Data was available on reasonable request and can be obtained from the corresponding author.

## Abbreviations

**ALT**: Alanine aminotransferase
BMI: Body mass index
CI: Confidence interval
DBP: Diastolic blood pressure
DR: Diabetic retinopathy
FBG: Fasting plasma glucose;
HDL-C: High-density lipoprotein cholesterol
HR: Hazard ratio
LDL-C: Low-density lipoprotein cholesterol
SBP: Systolic blood pressure
SD: Standard deviation
TBiL: Total bilirubin
TC: Total cholesterol
TG: Triglyceride
2hPG: Two-hour plasma glucose.

## Supplementary Materials

Supplementary Materials Appendix Table 1: the general characteristics of participants according to the quintiles of baseline total bilirubin (TBiL) levels. Appendix Table 2: the distribution (including mean, SD, median, and interquartile range) of baseline, follow-up, and change of TBiL levels (*μ*mol/L). Appendix Table 3: the HRs and 95% CI of diabetic retinopathy (DR) incidence according to baseline TBiL levels (*μ*mol/L) (excluding DR cases in the first two years) using the Cox model. Appendix Table 4: the HRs and 95% CI of DR incidence according to follow-up TBiL changes (*μ*mol/L) (excluding DR cases in the first two years) using the Cox model. Appendix Table 5: the HRs and 95% CI of DR incidence according to baseline TBiL levels (*μ*mol/L) (by different age groups, ≤80 yrs vs. >80 yrs) using the Cox model. Appendix Table 6: HRs and 95% CI of DR incidence according to follow-up TBiL changes (*μ*mol/L) (by different age groups, ≤80 yrs vs. >80 yrs) using the Cox model.
