# Supplementary material for: The U-Shaped Association between Bilirubin and Diabetic Retinopathy Risk: A Five-Year Cohort Based on 5323 Male Diabetic Patients
Source: J Diabetes Res. 2018 Oct 30;2018:4603087. doi: 10.1155/2018/4603087 (PMC6232811; doi:10.1155/2018/4603087)
Supplement: Supplementary Materials — Appendix Table 1: the general characteristics of participants according to the quintiles of baseline total bilirubin (TBiL) levels. Appendix Table 2: the distribution (including mean, SD, median, and interquartile range) of baseline, follow-up, and change of TBiL levels (μmol/L). Appendix Table 3: the HRs and 95% CI of diabetic retinopathy (DR) incidence according to baseline TBiL levels (μmol/L) (excluding DR cases in the first two years) using the Cox model. Appendix Table 4: the HRs and 95% CI of DR incidence according to follow-up TBiL changes (μmol/L) (excluding DR cases in the first two years) using the Cox model. Appendix Table 5: the HRs and 95% CI of DR incidence according to baseline TBiL levels (μmol/L) (by different age groups, ≤80 yrs vs. >80 yrs) using the Cox model. Appendix Table 6: HRs and 95% CI of DR incidence according to follow-up TBiL changes (μmol/L) (by different age groups, ≤80 yrs vs. >80 yrs) using the Cox model. [file 4603087.f1.doc]

Appendix Table1. General characteristics of participants according to baseline TBiL levels

| Characteristics | Quintiles of baseline TBiL levels | | | | |  |
| --- | --- | --- | --- | --- | --- | --- |
|  | Q1  (≤9.20) | Q2  (9.20-12.60) | Q3  (12.60-13.80) | Q4  (13.80-16.50) | Q5  (≥16.50) | P |
|  | n=1083 | n=1056 | n=1161 | n=976 | n=1047 |  |
| mean±SD |  |  |  |  |  |  |
| Age(yrs) | 79.28±8.08 | 78.70±8.43 | 78.56±8.55 | 78.73±8.07 | 78.18±8.73 | 0.047 |
| Duration(yrs) | 18.53±8.06 | 17.95±7.63 | 17.21±7.50 | 16.84±7.63 | 16.28±7.08 | 0.038 |
| Height(cm) | 169.37±5.09 | 169.81±5.40 | 169.31±5.51 | 170.06±5.40 | 169.59±5.54 | 0.008 |
| Weight(kg) | 72.11±8.07 | 72.57±8.77 | 73.25±8.86 | 72.59±8.65 | 72.43±8.21 | 0.030 |
| BMI(kg/m^2^) | 25.14±2.65 | 25.16±2.81 | 25.58±2.96 | 25.10±2.81 | 25.19±2.68 | 0.068 |
| SBP(mmHg) | 133.55±13.41 | 133.33±14.58 | 132.86±13.74 | 133.49±13.94 | 133.32±14.46 | 0.799 |
| DBP(mmHg) | 73.62±9.45 | 73.61±9.73 | 73.40±9.33 | 74.19±9.70 | 74.41±8.99 | 0.067 |
| Hb(g/L) | 130.62±17.83 | 135.31±16.87 | 138.35±16.53 | 138.12±17.20 | 140.86±17.52 | <0.001 |
| TC(mmol/l) | 4.64±1.12 | 4.79±1.06 | 4.70±0.98 | 4.87±1.05 | 4.78±1.03 | 0.086 |
| TG(mmol/l) | 1.74±1.21 | 1.69±1.16 | 1.64±0.95 | 1.65±0.97 | 1.64±1.04 | 0.180 |
| HDL-C(mmol/l) | 1.29±0.42 | 1.31±0.41 | 1.30±0.49 | 1.36±0.40 | 1.38±0.43 | <0.001 |
| LDL-C(mmol/l) | 2.65±0.87 | 2.70±0.86 | 2.61±0.70 | 2.75±0.88 | 2.67±0.85 | 0.102 |
| FPG(mmol/l) | 6.82±1.99 | 6.83±1.80 | 7.07±1.97 | 6.93±1.80 | 7.05±1.90 | 0.002 |
| 2hPG(mmol/l) | 9.15±2.94 | 9.21±2.71 | 9.21±2.76 | 9.31±2.89 | 9.50±3.00 | 0.031 |
| ALT(U/L) | 20.34±4.14 | 20.13±4.87 | 20.70±4.62 | 21.12±4.39 | 21.60±4.76 | 0.010 |
| baseline TBiL(μmol/L) | 7.48±1.72 | 10.97±0.71 | 12.92±3.67 | 14.70±0.84 | 19.54±3.07 | <0.001 |
| % |  |  |  |  |  |  |
| Education |  |  |  |  |  | 0.895 |
| ≤6 yrs | 65.2 | 65.4 | 65.3 | 65.9 | 65.5 |  |
| ≥7 yrs | 34.8 | 34.6 | 34.7 | 34.1 | 34.5 |  |
| Marriage status |  |  |  |  |  | 0.384 |
| Divorced / widowed | 13.0 | 14.8 | 10.4 | 12.6 | 12.7 |  |
| Married | 87.0 | 85.2 | 89.6 | 87.4 | 87.3 |  |
| Current smoking |  |  |  |  |  | 0.627 |
| Yes | 22.0 | 22.5 | 21.9 | 21.5 | 21.8 |  |
| No | 78.0 | 77.5 | 78.1 | 78.5 | 78.2 |  |
| Current alcohol drinking |  |  |  |  |  | 0.483 |
| Yes | 19.2 | 19.1 | 18.5 | 18.7 | 18.9 |  |
| No | 80.8 | 80.9 | 81.5 | 81.3 | 81.1 |  |
| Overweight/obesity |  |  |  |  |  | 0.258 |
| Yes | 66.6 | 67.4 | 72.2 | 65.6 | 69.9 |  |
| No | 33.4 | 32.6 | 27.8 | 34.4 | 30.1 |  |
| Hypertension |  |  |  |  |  | 0.690 |
| Yes | 60.9 | 63.0 | 62.9 | 63.7 | 61.5 |  |
| No | 39.1 | 37.0 | 37.1 | 36.3 | 38.5 |  |
| Dyslipidemia |  |  |  |  |  | 0.240 |
| Yes | 34.2 | 35.8 | 33.1 | 38.1 | 35.8 |  |
| No | 65.8 | 64.2 | 66.9 | 61.9 | 64.2 |  |
| Control of diabetes |  |  |  |  |  | 0.001 |
| Yes | 54.4 | 51.8 | 51.8 | 46.8 | 46.3 |  |
| No | 45.6 | 48.2 | 48.2 | 51.4 | 53.7 |  |

Appendix Table 2. The distribution of baseline, follow-up and change of TBiL levels (μmol/L)

| TBiL | Mean | SD | Median | IQR |
| --- | --- | --- | --- | --- |
| Baseline | 13.05 | 1.65 | 12.30 | 9.20-15.30 |
| Follow-up | 12.54 | 1.64 | 12.50 | 10.00-15.70 |
| Changes of TBiL | -0.66 | 1.93 | -0.73 | -3.30-2.10 |
| DR | -1.60 | 1.48 | -1.11 | -3.86-1.20 |
| NDR | -0.61 | 2.05 | -0.70 | -3.30-2.10 |

Appendix Table 3. HRs and 95% CI of DR incidence according to baseline TBiL levels (μmol/L) (excluding DR cases in the first two years)

| Variable type | HR*(95%CI) | *P* |
| --- | --- | --- |
| Continuous variable | 0.996(0.990-0.999) | 0.023 |
| Quintiles |  | 0.038 |
| Q1 | 1.941(1.243-3.032) |  |
| Q2 | 1.675(1.060-2.648) |  |
| Q3 | 1.00(Ref) |  |
| Q4 | 1.586(0.988-2.546) |  |
| Q5 | 1.707(1.075-2.711) |  |

*Adjusted for age, marital status, current smoking, current alcohol drinking, BMI, baseline Hb, ALT, baseline prevalence of hypertension and dyslipidemia, control of diabetes, duration of diabetes and follow- up TBiL changes(as continuous variable) in the model.*

Appendix Table 4. HRs and 95% CI of DR incidence according to follow-up TBiL changes (μmol/L) (excluding DR cases in the first two years)

|  | Variable type | HR*(95%CI) | *P* |
| --- | --- | --- | --- |
| Total population(n=5323) | Continuous variable | 0.973(0.947-0.998) | 0.038 |
|  | Categorical variable |  | 0.024 |
|  | ≤-2 | 1.394(1.045-1.858) |  |
|  | -2 to 2 | 1.00(Ref) |  |
|  | ≥2 | 0.885(0.615-1.274) |  |
| Among those with baseline TBiL level≤ 12.5μmol/L(n=2651) | Continuous variable | 0.956(0.923-0.990) | 0.012 |
|  | Categorical variable |  | 0.041 |
|  | ≤-2 | 1.436(1.032-2.121) |  |
|  | -2 to 2 | 1.00(Ref) |  |
|  | ≥2 | 0.834(0.633-0.954) |  |
| Among those with baseline TBiL level> 12.5μmol/L(n=2632) | Continuous variable | 0.985(0.940-0.992) | 0.518 |
|  | Categorical variable |  | 0.062 |
|  | ≤-2 | 1.478(0.980-2.229) |  |
|  | -2 to 2 | 1.00(Ref) |  |
|  | ≥2 | 0.924(0.490-1.743) |  |

*Adjusted for age, marital status, current smoking, current alcohol drinking, BMI, baseline Hb, ALT, baseline prevalence of hypertension and dyslipidemia, control of diabetes, duration of diabetes and baseline TBiL levels (as continuous variable) in the model.*

Appendix Table 5. HRs and 95% CI of DR incidence according to baseline TBiL levels (μmol/L) (by different age groups)

| Age groups | Variable type | HR*(95%CI) | *P* |
| --- | --- | --- | --- |
| ≤80 yrs | Continuous variable | 0.996(0.990-0.999) | 0.031 |
|  | Quintiles |  | 0.014 |
|  | Q1 | 2.094(1.995-4.002) |  |
|  | Q2 | 1.806(0.815-3.543) |  |
|  | Q3 | 1.00(Ref) |  |
|  | Q4 | 1.658(0.888-3.022) |  |
|  | Q5 | 1.883(1.046-3.388) |  |
| ＞80 yrs | Continuous variable | 0.995(0.989-1.000) | 0.050 |
|  | Quintiles |  | 0.195 |
|  | Q1 | 1.720(1.275-2.106) |  |
|  | Q2 | 1.649(1.174-2.004) |  |
|  | Q3 | 1.00(Ref) |  |
|  | Q4 | 1.515(0.887-2.344) |  |
|  | Q5 | 1.833(1.089-3.256) |  |

*Adjusted for age, marital status, current smoking, current alcohol drinking, BMI, baseline Hb, ALT, baseline prevalence of hypertension and dyslipidemia, control of diabetes, duration of diabetes and follow- up TBiL changes(as continuous variable) in the model.*

Appendix Table 6. HRs and 95% CI of DR incidence according to follow-up TBiL changes (μmol/L) (by different age groups)

| Age groups |  | Variable type | HR*(95%CI) | *P* |
| --- | --- | --- | --- | --- |
| ≤80 yrs | Total population(n=5323) | Continuous variable | 0.981(0.949-0.999) | 0.027 |
|  |  | Categorical variable |  | 0.023 |
|  |  | ≤-2 | 1.422(1.091-1.924) |  |
|  |  | -2 to 2 | 1.00(Ref) |  |
|  |  | ≥2 | 0.850(0.767-0.950) |  |
|  | Among those with baseline TBiL level≤ 12.5μmol/L(n=2651) | Continuous variable | 0.988(0.966-1.012) | 0.152 |
|  |  | Categorical variable |  | 0.042 |
|  |  | ≤-2 | 1.787(1.307-2.345) |  |
|  |  | -2 to 2 | 1.00(Ref) |  |
|  |  | ≥2 | 0.866(0.776-0.921) |  |
|  | Among those with baseline TBiL level> 12.5μmol/L(n=2632) | Continuous variable | 0.953(0.909-1.000) | 0.050 |
|  |  | Categorical variable |  | 0.049 |
|  |  | ≤-2 | 1.159(0.799-1.684) |  |
|  |  | -2 to 2 | 1.00(Ref) |  |
|  |  | ≥2 | 0.842(0.551-1.356) |  |
| ＞80 yrs | Total population(n=5323) | Continuous variable | 0.950(0.917-0.984) | 0.004 |
|  |  | Categorical variable |  | 0.028 |
|  |  | ≤-2 | 1.541(1.182-2.050) |  |
|  |  | -2 to 2 | 1.00(Ref) |  |
|  |  | ≥2 | 0.858(0.770-0.947) |  |
|  | Among those with baseline TBiL level≤ 12.5μmol/L(n=2651) | Continuous variable | 0.947(0.901-0.998) | 0.049 |
|  |  | Categorical variable |  | 0.003 |
|  |  | ≤-2 | 1.998(1.345-3.002) |  |
|  |  | -2 to 2 | 1.00(Ref) |  |
|  |  | ≥2 | 0.887(0.779-0.981) |  |
|  | Among those with baseline TBiL level> 12.5μmol/L(n=2632) | Continuous variable | 0.953(0.911-0.996) | 0.032 |
|  |  | Categorical variable |  | 0.251 |
|  |  | ≤-2 | 1.101(0.798-1.563) |  |
|  |  | -2 to 2 | 1.00(Ref) |  |
|  |  | ≥2 | 0.892(0.612-1.314) |  |

*Adjusted for age, marital status, current smoking, current alcohol drinking, BMI, baseline Hb, ALT, baseline prevalence of hypertension and dyslipidemia, control of diabetes, duration of diabetes and baseline TBiL levels (as continuous variable) in the model.*
